# A Critical Analysis of the Opportunities and Challenges of Phage Application in Seafood Quality Control

**DOI:** 10.3390/foods13203282

**Published:** 2024-10-16

**Authors:** Jun Yan, Zhenghao Guo, Jing Xie

**Affiliations:** 1 College of Food Science & Technology, Shanghai Ocean University, Shanghai 201306, China; jyan@shou.edu.cn (J.Y.); gzh1214160041@163.com (Z.G.); 2 Laboratory for Quality and Safety Risk Assessment of Aquatic Products in Storage and Preservation of Ministry of Agriculture and Rural Affairs, Shanghai Ocean University, Shanghai 201306, China; 3 National Experimental Teaching Demonstration Center for Food Science and Engineering, Shanghai Ocean University, Shanghai 201306, China; 4 Shanghai Professional Technology Service Platform on Cold Chain Equipment Performance and Energy Saving Evaluation, Shanghai Ocean University, Shanghai 201306, China

**Keywords:** phage, production, biocontrol, detection, seafood

## Abstract

Seafood is an important source of food and protein for humans. However, it is highly susceptible to microbial contamination, which has become a major challenge for the seafood processing industry. Bacteriophages are widely distributed in the environment and have been successfully used as biocontrol agents against pathogenic microorganisms in certain food processing applications. However, due to the influence of environmental factors and seafood matrices, using bacteriophages for commercial-scale biocontrol strategies still faces some challenges. This article briefly introduces the current processes used for the production and purification of bacteriophages, lists the latest findings on the application of phage-based biocontrol in seafood, summarizes the challenges faced at the current stage, and provides corresponding strategies for solving these issues.

## 1. Introduction

Seafood stands as a rich source of high-quality protein and essential trace elements crucial for human health, making it a highly preferred nutritional component in daily diets. It is estimated that by 2030, the per capita supply of seafood will reach 21.4 kg [1]. However, seafood is susceptible to microbial contamination during harvesting, handling, storage, processing, and distribution. Specific spoilage bacteria include *Pseudomonas*, *Acinetobacter*, and *Serratia* [2]. Additionally, pathogenic bacteria such as *Escherichia coli*, *Listeria monocytogenes*, *Clostridium botulinum*, *Staphylococcus aureus*, *Salmonella typhimurium*, *Vibrio vulnificus*, *Vibrio cholerae*, and *Vibrio parahaemolyticus* can also infect humans through the seafood supply chain, causing varying degrees of impact on human health and safety [3]. Consequently, it is imperative to implement effective microbial control measures to guarantee the quality and safety of seafood.

Traditional processing methods for seafood, including smoking, dehydration, fermentation, canning, and salting, significantly alter its physicochemical properties. Such alterations may encompass protein denaturation, a reduction in vitamin content, color variations, and disturbances to the natural microbiota [4]. To address the issues associated with traditional processing methods, researchers have developed various non-thermal processing technologies (high hydrostatic pressure, pulsed electric fields, cold plasma, pulsed light, irradiation, ozone treatment, etc.) for treating seafood [5,6,7]. These technologies not only extend the shelf life of seafood but also preserve its natural color, flavor, texture, nutritional value, and functional qualities. Although these technologies have promising applications, they still face significant challenges in large-scale implementation and require specialized equipment and well-trained personnel.

Bacteriophages, viruses targeting specific bacteria, provide a highly precise and selective approach for controlling microbial contamination in seafood. These viruses have a specific affinity for only their designated bacterial targets, leaving beneficial bacteria and host cells unharmed. With a low production cost and the ability to complement other antimicrobial methods, bacteriophages enhance the overall bactericidal effect. Their incorporation into food packaging materials ensures continuous protection during storage [8]. As the preference for raw or lightly cooked seafood grows among consumers [9], bacteriophages emerge as a promising solution. Studies confirm that they do not alter the sensory qualities of seafood and are safe for human consumption, aligning perfectly with practical application requirements [5].

This review focuses on the application of bacteriophages in the biocontrol of seafood. Initially, methods for isolating and purifying bacteriophages are outlined, along with a brief discussion of their respective advantages and disadvantages. Subsequently, recent laboratory research on the applications of bacteriophages in seafood is reviewed. Finally, the challenges in standardizing bacteriophage applications are discussed, and potential strategies to overcome these issues are proposed. The aim is to provide theoretical support for the wider application of bacteriophages in the seafood industry.

## 2. Overview of Phage

The discovery of phages is credited to Frederick Twort and Felix D’Herelle. While observing bacterial colonies on culture medium, Twort noticed a glassy transformation and hypothesized the presence of a small virus that inhibited bacterial growth. Meanwhile, Felix D’Herelle identified a microbe with an antagonistic effect on bacteria, which he named a phage [10]. The morphological structure of phages was not recognized until 1940, when they were observed using an electron microscope [11]. In 2006, the U.S. Food and Drug Administration (FDA) approved phage products (ListShield^TM,^, Intralytix, Baltimore, MD, USA) for use in food processing. Since then, research on the use of phage products in different foods and at various stages of processing has grown [12].

Phages utilized in production applications are strictly limited to virulent phages, which generally follow a life cycle consisting of five stages: adsorption, penetration, replication, maturation, and lysis. Adsorption represents a crucial step in the phage–host interaction. Phages initially bind non-specifically to host cell surface receptors before progressing to specific binding [13]. Typically, phage adsorption generally comprises three stages: initial contact, reversible binding, and irreversible binding [14]. During the initial contact phase, phages, unable to move independently, rely on Brownian motion and diffusion to facilitate random collisions with the host cell surface [15]. Consequently, as the concentration of both phages and host cells escalates, the likelihood of collision and adsorption rate also increases. Certain host cells possess flagella enabling independent movement, significantly elevating the collision probability. In reversible adsorption, the phage interacts with primary receptors on the host surface without undergoing permanent structural changes that allow desorption. In contrast, irreversible adsorption involves specific binding between the phage and the secondary receptor, with an increasing interaction force that is difficult to break [16]. Studies on the adsorption process in phages, such as T4, have shown that long-tail fibers initially reversibly adsorb to specific receptors, followed by the irreversible adsorption of short-tail fibers to LPS molecules [17].

After adsorption, the bacteriophage swiftly injects its nucleic acids into the bacterial cell, manipulating the host’s metabolic machinery to replicate the phage nucleic acids in large quantities and synthesize the proteins necessary for viral assembly. For progeny bacteriophages to be released, the host bacterium must undergo lysis, which can be categorized into multi-gene lysis and single-gene lysis. Large dsDNA phages, such as Caudovirales, possess multiple lysis proteins, including holins, endolysins, and spanins, which target the cytoplasmic or inner membrane (IM), peptidoglycan (PG), and outer membrane (OM), respectively, along with various proteins that regulate the lysis process. In contrast, lytic ssDNA and ssRNA phages utilize a single lysis protein to induce cell lysis without enzymatically degrading the PG [18].

## 3. Phage Production

### 3.1. Phage Isolation

Compared to traditional antimicrobials, the ease of isolating new types of bacteriophages is a major advantage of phage-based biocontrol [19]. In the environment where the host bacteria live, it is easy to isolate bacteriophages with lytic activity. Phage isolation has long followed the method proposed by Felix d’Herelle. In simple terms, this involves co-cultivating the host bacteria with the collected environmental sample at an appropriate temperature for a period of time (usually overnight), followed by centrifugation and filtration. The sample is then directly spotted onto a plate with host bacteria to observe whether any plaques are formed [20]. Subsequently, the biological properties of the phage, including the length of the latent period, burst size, genome sequence, and other factors, must be characterized to determine the phage’s potential for application.

### 3.2. Phage Cultivation 

Phage cultivation is a key step in phage production, primarily aimed at generating large quantities of phages. To maximize the amount of phages, a fine balance between the dynamics of phages and host populations must be established [21]. The key factors influencing phage production include the host bacteria’s specific growth rate, cell life cycle stage, metabolic activity, and density of phage receptors, as well as the phage’s adsorption rate, latent period, burst size, and multiplicity of infection [22,23]. After defining the operating parameters, the bioreactor is ready for production. Bioreactors for phage production can be operated in batch, semi-continuous, and continuous modes and should be run under optimal operational and infection conditions to maximize the phage titer [21].

Batch cultivation is a commonly used operational mode that is simple to implement, highly controllable, and capable of producing high-titer bacteriophages (10^10^–10^16^ PFU/mL). In this mode, the host bacteria and bacteriophages are cultured to a defined concentration before being simultaneously introduced into the same fermenter at an appropriate multiplicity of infection (MOI). Until harvest, the growth conditions of the host bacteria and phages are governed by population dynamics [24]. As early as the 1950s, research on the batch cultivation of bacteriophages had been conducted. Lunan and others used batch cultivation to grow T7 bacteriophages with an MOI of 0.1, ultimately achieving a titer of 10^11^ PFU/mL in the final product [25]. Product variation between different batches is a significant drawback of batch production, which can impact subsequent processes. There are other drawbacks to batch production, such as the yield being limited by the size of the fermenter, resulting in relatively low output. Additionally, after each batch is harvested, the fermenter must undergo thorough cleaning and disinfection, leading to longer preparation times and requiring significant labor [26]. 

Continuous bacteriophage production effectively addresses challenges of low productivity, inconsistent product quality, and high production costs associated with batch production. In continuous phage production, fresh culture medium is continuously supplied, while waste medium containing host bacteria and phages is continuously withdrawn. In this mode, the extended co-cultivation of host bacteria and phages may lead to genetic mutations, making single-stage continuous bioreactors (e.g., chemostats or turbidostats) less suitable for large-scale phage production [24]. To reduce the occurrence of mutations, two-stage continuous reactors (cellstats) have been developed. These consist of two continuously stirred tanks of different sizes and a final storage tank [27]. Host bacteria are cultured in the larger tank, and their growth is adjusted by the injection of fresh medium to keep them in the exponential growth phase, which is conducive to phage infection. Since the host bacteria are cultured alone in the first fermenter, the likelihood of phage-resistant bacteria emerging is significantly reduced [23]. The host bacteria culture is then transferred to the second reactor for infection. To ensure successful bacteriophage infection, the injection rate of the host bacteria culture must match the phage infection rate; otherwise, more phages may leave the fermenter before infecting the bacteria [21].

The semi-continuous operation mode combines the advantages of batch production and continuous operation. It allows for the efficient production of high-titer bacteriophages with stable quality while also shortening preparation time and reducing the required footprint [21]. It consists of two bioreactors with self-circulation. In this operation mode, the host bacteria are first grown in the first bioreactor until they approach the stationary phase, at which point half of the culture medium is immediately transferred to the second bioreactor, and fresh medium is added. This keeps the host bacteria in the exponential growth phase. In the second bioreactor, the host bacteria are infected by bacteriophages. After a specified cultivation time, most of the bacteriophages are harvested, and fresh medium is added, leaving only a small portion of the bacteriophages to participate in the next round of infection [24]. Throughout the production process, except for the transfer of host bacteria and bacteriophages, the two reactors operate independently, significantly reducing the risk of contamination. Additionally, the co-cultivation time of the host bacteria and bacteriophages is greatly shortened in this mode, reducing the likelihood of bacteriophage-resistant bacteria emerging [23].

### 3.3. Phage Purification

To obtain phage that meets expectations, phage purification is required to remove cellular debris, genomic DNA, RNA, proteins, and endotoxins (LPS) that remain during phage culture. At present, the main methods for obtaining bacteriophages include precipitation, chromatography, tangential flow filtration, and aqueous two-phase extraction.

Precipitation is a relatively inexpensive and easy-to-use technique for phage isolation, which is very effective for concentration and purification of phage. Precipitation is often used to prepare phage stocks in the laboratory and is capable of removing unprecipitated low-molecular-weight impurities. Among them, the chemical precipitation method involves the addition of various chemical reagents to the phage suspension to saturate the suspension and precipitate the phage, thereby obtaining phages. Branston et al. used the polyethylene glycol-salt precipitation method to recover M13 phage from clarified media under three different conditions (i. 4% (*w*/*v*) of PEG 6000, ii. 2% (*w*/*v*) PEG 6000 with 135 mM of NaCl, and iii. 2% (*w*/*v*) PEG 6000 with 25 mM of MgSO_4_), and the recovery rate was over 90% in all cases [28]. This method removed most of the DNA impurities, with 97% of the initial DNA retained in the supernatant, and removed a significant amount of protein with a purification factor of over 250. Isoelectric precipitation is also a commonly used method for separating phage. It involves neutralizing the net charge of the phage by adding acid or base so that the pH is close to the isoelectric point of the phage and aggregate. Dong et al. successfully isolated phage M13 from cell supernatant with high recovery (98.4% to 91.4%) by adjusting the isoelectric pH near 4.2. However, the precipitation method is easily affected by temperature, pH, protein concentration, and precipitant. It is commonly used for laboratory phage isolation but rarely for large-scale phage purification [29].

Chromatography is also a commonly used downstream separation technique for bioengineering, which helps to obtain products with high purity and recovery and is easily scalable, providing a good platform for large-scale production. Different interaction modes have been used for phage purification, including ion exchange, size exclusion, etc. Monjezi et al. successfully isolated M13 phage from clarified lysates with 79% recovery using ion exchange chromatography filled with a conventional strong anion exchange resin and N^+^(CH_3_)_3_ ligand, eluted with citrate buffer [30]. Zakharova et al. used size exclusion chromatography using a 30 cm long column filled with Sephacryl™ S-500 resin (Amersham Biosciences, Piscataway, NJ, USA) to remove recalcitrant proteins and low molecular weight impurities from a PEG-purified phage preparation of M13 phage, which did not significantly reduce the viability of the phage and effectively removed most of the impurities [31].

Tangential flow filtration (TFF) is a commonly used technique for the separation and concentration of target products. Its main principle is to flow the liquid tangentially along the membrane surface to separate and concentrate the target substances. The choice of membrane plays a crucial role in the separation effectiveness of this method. Different membrane materials and pore sizes can affect the efficiency and quality of the filtration. In addition, transmembrane pressure and cross-flow velocity are key parameters that need to be considered during the process. Castro-Mejía et al. used tangential flow filtration to extract phages from fecal samples and found that, compared to a literature-adapted protocol, this method had a higher recovery rate, producing up to 16 times more phage particles and up to 68 times more phage DNA per volume [32]. 

**Figure 1:**
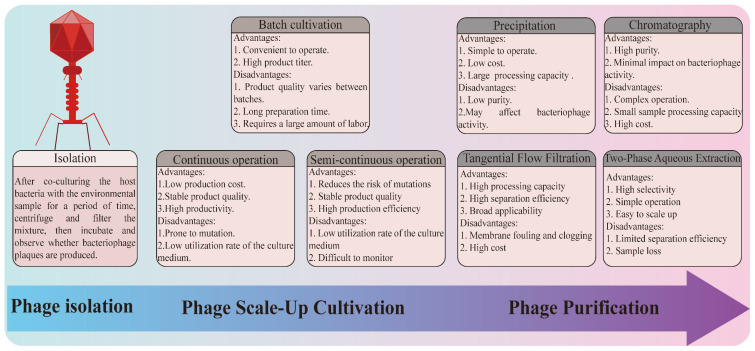
Flow chart of phage production.

Aqueous two-phase extraction (ATPE) is a widely used liquid–liquid extraction technique for biological separation and purification. It utilizes two water-soluble polymers to form two immiscible aqueous phases, in which different biomolecules distribute between the phases based on their hydrophilicity, molecular weight, and other properties. Gonzalez-Mora et al. tested several PEG salt systems for the purification of phage M13 from crude lysates and achieved 83% recovery when using 17.2% PEG 400 and 15.5% potassium dihydrogen phosphate, with most of the phage being assigned to the top phase [33]. Clavijo et al. found that PEG 8000-potassium phosphate resulted in 55-fold bottom phase partitioning and a 100-fold reduction in protein impurities [34]. However, phage partitioning in a dual aqueous phase system with high solute concentrations can induce osmotic shock while producing nucleic acid-free ghost phages that are unable to infect the host (Figure 1).

## 4. Applications of Phage in Seafood

### 4.1. Biocontrol

Many phages are already being used in the laboratory to prevent and control common pathogenic and spoilage bacteria. As such, they are expected to become a mainstay of biodefense in the seafood processing industry (Table 1).

*Vibrio* is a genus of Gram-negative, curved rod-shaped bacteria ubiquitously distributed in marine and coastal ecosystems. These bacteria not only infect marine organisms, leading to considerable economic losses (estimated at USD 1 billion), but also infect humans, particularly those with compromised immune systems [45]. Symptoms of Vibrio infections can vary, ranging from mild gastrointestinal disturbances to severe wound infections and, in extreme cases, septicemia. Notable pathogenic species of the *Vibrio* genus include *V. parahaemolyticus*, *V. cholerae*, and *V. vulnificus* [46]. *V. parahaemolyticus* is a Gram-negative bacterium commonly found in various aquatic environments [47]. It is an opportunistic pathogen known to infect aquatic animals through multiple virulence factors, causing significant losses to the aquaculture industry. Phage therapy has shown promising results in aquaculture [48,49]. Ren et al. (2019) isolated two strains of *V.  parahaemolyticus* phages, PVP 1 and PVP 2, from the sewage of an aquaculture farm. They combined the lyophilized powder of both phages with feed and fed it to sea cucumber. This study found that the phage treatment group with MOIs of 1 and 100 showed increased protection against *V.  parahaemolyticus* VP-ABTNL compared with the antibiotic treatment group. There was no significant difference in weight gain, feeding rate, or food conversion rate between the two phage treatment groups and the control group without phage [37]. You et al. (2021) isolated a lytic *V.  parahaemolyticus* phage strain, VPT 02, from oyster meat. They found that it also had a lytic effect on antibiotic-resistant *V.  parahaemolyticus*. Treatment of brine shrimp infected with *V.  parahaemolyticus* using VPT 02 with an MOI of 10 significantly increased the survival rate of brine shrimp from 16.7% to 46.7% compared to the untreated control group [36]. Lee et al. (2023) isolated and identified *V.  parahaemolyticus* phage VPG01 and investigated its inhibitory effect on *V. parahaemolyticus* on the flesh of dental turbot. The test results showed that *V. parahaemolyticus* was rapidly removed from the fish flesh treated with phage with a MOI of 10 at 6 h, reaching a pathogen load below the detection limit [9]. *V. cholerae* is a prevalent foodborne pathogen transmitted via contaminated water or food sources. Upon entering the human body, it colonizes the small intestine and secretes cholera toxin, resulting in acute watery diarrhea and vomiting. If untreated, severe cases may lead to fatality due to dehydration and electrolyte imbalance. *V. cholerae* is predominantly classified into O1 and O139 serogroups, with the O1 serogroup being the primary driver of global cholera outbreaks. Ahmadi et al. investigated the combined effect of virulent bacteriophages and high hydrostatic pressure (HHP) in controlling *V. cholerae* in salmon and mussels. The results demonstrated that the application of bacteriophages alone reduced *V. cholerae* counts by 1.2 log10 CFU/g. However, the combination with HHP significantly enhanced pathogen inactivation, resulting in a reduction in *V. cholerae* counts ranging from 2.6 to 3.3 log10 CFU/g [50]. *V. vulnificus* is a Gram-negative, halophilic, curved rod-shaped bacterium predominantly found in warm coastal waters. It is among the most virulent species of *Vibrio*, primarily infecting humans via the consumption of contaminated seafood, particularly raw oysters, or through wound exposure to contaminated seawater. Kim et al. investigated the efficacy of the virulent bacteriophage VVP001 in controlling *V. vulnificus* in fresh abalone. The results demonstrated that at a multiplicity of infection (MOI) of 10⁶, *Vibrio vulnificus* counts decreased by 2.51 log CFU/mL, while at an MOI of 10⁵, the counts declined by 2.06 log CFU/mL. VVP001 consistently inhibited the growth of *V. vulnificus* for up to 8 h [39]. Pelon et al. reported that the combination of oyster extracts with bacteriophages resulted in a reduction in *V. vulnificus* counts in *Crassostrea virginica* from 10⁶ to 10^1^ CFU/mL after 18 h of incubation at 4 °C [51].

*L. monocytogenes* is a prevalent pathogen in aquatic products due to its robust survival ability, enabling it to grow and reproduce under harsh conditions such as low temperatures, high salt, and acidity [52]. The FDA approved ListShield™ and Listex P-100™ phage preparations for controlling *L. monocytogenes*. Banos et al. (2016) showed that combining intestinal microbiota AS-48 with *L. monocytogenes*-lysed phage P100 effectively controlled *L. monocytogenes* in cod and salmon [53]. Gündüz and Öztürk (2021) found that adding Listex P100™ into a sodium alginate-based film and applying it directly to the surface of smoked rainbow trout resulted in an increased phage titer of 8.54 log PFU/g on day 7 of storage, suggesting its effectiveness against *L. monocytogenes* growth [54]. Zhou et al. reported the isolation of a novel *L. monocytogenes* bacteriophage, designated SH 3-3, from a food processing plant, which demonstrated the ability to inhibit *L. monocytogenes* biofilms at the minimum effective bactericidal concentration. When applied to raw salmon, the *L. monocytogenes* count decreased by 4.54 log within a period of 72 h [41].

*Salmonella typhimurium* is a prevalent foodborne pathogen capable of causing acute gastroenteritis. In individuals with compromised immune systems or underlying health conditions, *S. typhimurium* can result in more severe infections, including septicemia. Xu et al. evaluated the efficacy of bacteriophage SLMP1 in reducing *S. typhimurium* on contaminated raw salmon filets and scallops. The results demonstrated that during storage at 4 °C, bacteriophage SLMP1 was able to reduce *Salmonella* counts in both inoculated samples to below the detection limit or maintain them at low levels [43]. Pereira et al. investigated the potential application of phages phSE-2, phSE-5, and the phage mixture phSE-2/phSE-5 in reducing *S. typhimurium* concentrations during the purification process of naturally and artificially contaminated shellfish. At a multiplicity of infection (MOI) of 0.1, the inactivation rate of the single phage suspension was greater than that of the phage mixture. However, in naturally contaminated conchs, after 6 h of treatment, the reductions in bacterial concentrations achieved with both the single phage suspension and the phage mixture phSE-2/phSE-5 were comparable, approximately 0.7–0.9 log CFU/g. In the case of artificially contaminated conchs purified with phage phSE-5 in a recirculating seawater system, the Salmonella concentrations decreased by 0.9 and 2.0 log CFU/g after 4 and 6 h of treatment, respectively. In contrast, when the purification process was conducted without phage treatment, only a 1.1 log CFU/g reduction in Salmonella was achieved after 6 h of treatment [55].

*Shewanella putrefaciens* is a Gram-negative, motile bacterium that is widely distributed in nature and is known to be a specific spoilage bacterium in aquatic products. Its ability to adapt to low temperatures allows it to grow and multiply in chilled aquatic products, making it the dominant spoilage bacterium. Additionally, *S. putrefaciens* can produce bioamines such as putrescine and cadherine, as well as odors such as trimethylamine and H_2_S, and cause surface slime, loose muscle, and quality deterioration of aquatic products. Yang et al. (2019) selected three phages (SppYZU01, SppYZU05, and SppYZU06) from four isolated and identified *S. baltica* phages and six *S. putrefaciens* phages based on host range and lytic activity. These three phages were combined to form a phage cocktail named SPMIX3-156. When the phage cocktail was separately inoculated into catfish broth stored at 25 °C and 4 °C, it was observed that the growth of Shewanella was inhibited [56]. Kong et al. conducted a preservation experiment on refrigerated turbot using the *S. putrefaciens* phage Spp001. The results showed that the phage had a significant preservative effect, which was markedly better than the control group that was not treated with the phage. Based on its preservative effect, the *S. putrefaciens* phage Spp001 can be fully utilized as a substitute for other preservatives in the preservation of refrigerated turbot [57].

*Pseudomonas* is ubiquitous in soil, water, and food processing plant environments, where it metabolizes to produce a plethora of aldehydes, ketones, esters, and volatile products with unpleasant odors [58]. *Pseudomonas fluorescens* and *Pseudomonas putida*, dominant spoilage bacteria in aquatic products, exhibit strong biofilm formation capabilities, enhancing the colonization and adhesion of pathogenic bacterial biofilms. Chu et al. evaluated the preservative effect of the virulent phage PrH-181 on large yellow croaker stored under aerobic refrigeration at 4 °C. Experimental results indicated that treatment with a phage titer of 10⁶ PFU/mL achieved the most effective results, extending the storage time by approximately 10 days compared to untreated fish under the same conditions [59]. Pang et al. used the dominant bacterium SSO-C02 as the host to isolate the *Pseudomonas* phage SSO-C02P1 through the double-layer agar plate method. When applied to clean filets of crisp grass carp, the spoilage endpoint was extended by three days [60].

**Figure 2:**
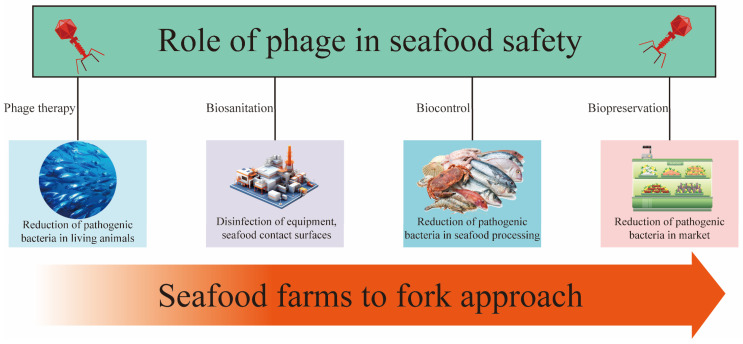
Uses of the phage treatment in the seafood production chain.

With growing global concern over food safety and public health, phages offer a promising biocontrol strategy to mitigate the spread of bacterial diseases via food. For example, seafood is susceptible to bacterial contamination during different stages of harvesting, transport, processing, and distribution. Phages can specifically target and eliminate pathogens during these stages, thereby reducing the risk of bacterial contamination in food. This approach not only safeguards consumer health but also helps mitigate economic losses associated with bacterial contamination (Figure 2).

### 4.2. Detection 

**Figure 3:**
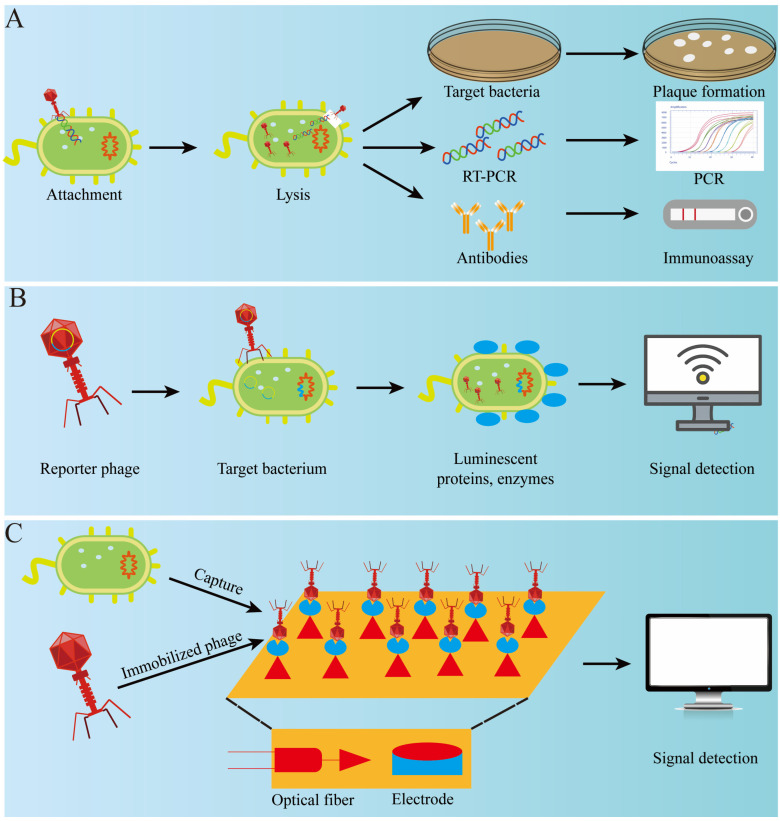
Principles of phage-based pathogen detection. (**A**) Methods dependent on the infection and metabolic activity or lysis of the bacteria cell. (**B**) Fundamental principles of pathogen detection using reporter phages. (**C**) Fundamental principles of phage-based biosensor detection.

Detection of bacteria in seafood can be challenging due to complex matrices, which require extensive pre-treatment and lead to low sensitivity and reproducibility. Traditional culture-based detection methods are accurate but time-consuming and labor-intensive. Therefore, developing simple, rapid, accurate, and cost-effective pathogen detection methods for cloudy or complex food matrices is essential. Due to its high host specificity, phage can be used for the rapid detection of bacteria and is suitable for various detection formats. Here, we will briefly describe several phage-based methods used for bacterial detection (Figure 3).

#### 4.2.1. Phage Amplification Detection

Phages are highly specific, making them useful for determining the viability of bacteria. The phage amplification assay (PAA) is a method used to detect pathogenic bacteria using virulent phages. These phages infect and quickly lyse host bacteria, releasing large quantities of progeny phages that form a plaque in a double plate, indicating the presence of host cells. Ding et al. (2023) used the LSA2311 phage to detect Staphylococcus aureus in 6.5 h [61]. With qPCR, the detection time was reduced to 3.5 h. Although the phage-based microbiological detection method is simple, effective, and inexpensive, it requires certain conditions, such as a total number of phages and infection time, and the method is often time-consuming. Therefore, this method is commonly used in some small-scale food processing plants.

#### 4.2.2. Phage-Based Immunological Detection

Immunological methods, such as enzyme-linked immunosorbent assays (ELISAs), rely on the specific reaction of antigens and antibodies. However, issues with batch-to-batch instability, large molecular mass, and high purification costs in antibody production have limited the development of these methods. Instead, phages and their structural components can be used to effectively overcome these problems. Stambach et al. (2015) developed a surface-enhanced Raman spectroscopy–lateral flow immunochromatography method for the detection of *L. monocytogenes* using A511 phage instead of the original primary antibody. This approach combined the phage with a Raman reactive dye, shortening the detection time from 8 h to 2 h [62].

#### 4.2.3. Reporter Phage Detection 

Reporter phage detection, also known as phage-based molecular biology detection, involves genetically engineering phages to encode fluorescent chromogenic or detectable genes such as green fluorescent protein gene (gfp), alkaline phosphatase, luciferase (lux and luc), and β-galactosidase gene (lacZ) [63]. Kim et al. (2017) designed a reporter phage, phiV10Lux, which could produce bioluminescence that corresponded to the number of *E. coli* present for the detection of *E. coli* O157:H7 in food samples. The detection limit was 1 CFU/mL within just 40 min [64]. 

#### 4.2.4. Phage-Based Optical Detection 

ATP can fluoresce when exposed to firefly luciferase, which is encoded by the eukaryotic luc gene. When phage lyses bacteria and releases intracellular ATP, energy is released from the ATP with the help of luciferase, producing fluorescence proportional to the ATP content. This method allows inference of the total number of colonies. Miyanaga et al. (2006) improved this method based on the principle that adenylate kinase (AK) can catalyze the conversion of ADP to ATP. AK released from phage lysis of bacteria converted the ADP added to the sample to ATP, which improved the luminescence intensity of the sample and was able to detect *E. coli* with less than 10^3^ CFU/mL within 1 h [65].

The nicotinamide adenine dinucleotide (NADH) content in microbiota cells at specific growth and metabolism stages was relatively stable and positively correlated with the number of microorganisms in food. When bacteria die, intracellular enzymes break down NADH. Using potent phages to lyse host bacteria releases intracellular NADH, which can be used in oxidoreductase and fluorescence. The total number of colonies can be calculated by measuring the fluorescence intensity under the action of holoenzyme. Roach et al. (2015) successfully detected Klebsiella by phage-mediated NADH bioluminescence [66].

Chemiluminescent reagents can chemically covalently bind or physically adsorb to some groups of cells, enabling detection of host bacteria through their luminescence characteristics. With the development of science and technology, commercial fluorescent reagents such as YOYO-1, SYBR, and SYTO have been gradually developed. Due to their high affinity with nucleic acid, these fluorescent reagents have replaced traditional fluorescent dyes to a certain extent. Goodridge (2007) detected *E. coli* O157:H7 with a detection limit of 100 CFU/mL [67].

#### 4.2.5. Phage-Based Biosensor Detection

Biosensors integrate multiple disciplines and offer low-cost, highly sensitive, and easy-to-use sensing technology that finds vast applications in biomedicine and the food industry, among others. The molecular recognition element is a vital component of any biosensor. Phage has gained attention as a new recognition element due to its high specificity, wide prevalence, easy accessibility, and stable nature. Zhou et al. (2022) developed an electrochemical sensor for the detection of *E. coli* O157:H7 with phage EP01 as the recognition element. The sensor yielded results within 30 min and had a detection range of 10^2^ to 10^7^ CFU/mL, with a limit of detection of 11.8 CFU/mL [68]. Similarly, Wang et al. (2023) developed a colorimetric immunosensor for detecting *V.  parahaemolyticus* by utilizing the high specificity of phage nbs and the optical properties of AuNPs. The visual detection limit of the sensor is 10^4^ cfu/mL, the quantitative detection limit is 10^3^ cfu/mL, and the detection time is less than 100 min [69].

## 5. Limitations of Phage Application 

Phages have been extensively studied and applied in the food industry. However, since food is closely related to human life and health, ensuring the safety of phage applications in the food industry is of paramount importance. This section will describe the current situation, challenges, and potential solutions for phage application in the food industry (Table 2).

### 5.1. Application Stability

Bacteriophages are composed of a protein shell and nucleic acids, making them susceptible to environmental factors that can lead to protein misfolding, aggregation, denaturation, and potential internal structural damage, resulting in the loss of activity [80]. Studies have found that factors such as temperature, pH, ion concentration, seafood matrix, and duration of UV exposure all affect the stability of bacteriophages [81]. Temperature is a key factor affecting bacteriophage activity. When exposed to non-optimal temperatures, the latent period of bacteriophages is extended, and only a few bacteriophages participate in proliferation [82,83]. The temperature range that bacteriophages can tolerate varies significantly depending on the climate of the location from which they were isolated and collected. Similarly, the pH and ion concentration range that bacteriophages can tolerate are typically closely aligned with the environment of their host. UV exposure may alter the genetic material of bacteriophages or degrade their proteins, resulting in the formation of photoproducts like cyclobutene pyrimidine dimers [84]. The unpredictability of these factors in practical applications has created a huge obstacle to the widespread use of phages. Phage encapsulation is the primary means of eliminating the effects of external environmental factors and maintaining phage stability. For example, it has been shown that phage titres did not change significantly after three months of storage at 4 °C and 22 °C using 1% alginate-embedded phage [85].

### 5.2. Complete Application Security

Resistance genes typically reside in a number of mobile genetic elements and can be transferred between bacteria via horizontal gene transfer, which is one of the reasons for the increasing spread of antibiotic resistance in food-borne bacterial pathogens [86]. Mild phages integrate their DNA into the host bacterial genome during proliferation, which would facilitate the spread of antibiotic resistance genes (ARGs). For example, phage EC10 transfers the resistance gene *bla* from *E. coli* NBRC 12713 KEN1 to *E. coli* C600RK2/HB101/NBRC 12713/W3110 [87], while phage P24 transfers the resistance gene *bla* from *Salmonella heidelberg* S25 to *S. typhimurium* MZ1262 [88]. Moreoverwhich is one of the reasons for the increasing spread of antibiotic resistance in food-borne bacterial pathogens (e.g., sewage, ocean, and soil), carry many ARGs that can be transferred to host bacteria by phages through transduction. Therefore, the role of phages in the spread of environmental resistance genes should not be underestimated [89]. 

### 5.3. Public Acceptance 

In recent years, consumers have increasingly preferred green and natural products over antibiotic-treated and chemically treated foods [90]. This trend has contributed to the increased use of phages in food. However, despite their potential benefits, the knowledge of phages among the public remains limited, which significantly hinders their widespread use in the food industry. Therefore, enhanced professional education of the public and food processing practitioners, as well as increased public awareness of the safety and efficacy of phage application, are priority options to promote the wider use of phages [90]. Phages are ubiquitous and can be found in various environments, including the ocean, the atmosphere, and living organisms. They also play important roles in maintaining microbial community structures in the human body, such as in the mouth, blood, and gut [91]. Furthermore, researchers have evaluated the safety of oral phage preparations in volunteers of different ages, confirming that oral administration of a certain amount of phage preparations has no adverse effects on human health [92]. Numerous food-grade phage formulations have been developed, including Listex™ P100, which was approved by the FDA in 2006 for use in ready-to-eat food and poultry products to prevent *L. monocytogenes* contamination in meat and cheese products. Listex™ P100, developed by EBI Food Safety, is an effective method of preventing *L. monocytogenes* contamination [93]. Nine phage formulations have been certified as GRAS (Generally Regarded as Safe), and two are in the process of being certified. Therefore, consumers can safely choose phage-treated foods already on the market.

### 5.4. Resistance to Bacteriophage

Bacteria and bacteriophages have co-evolved for billions of years, and bacteria have developed robust phage defense systems [94]. Based on the mechanisms by which bacteria resist phage infection, bacterial phage resistance can be categorized into five types: blocking phage adsorption, preventing phage DNA injection, inhibiting DNA replication, abortive infection, and preventing phage assembly and release [95]. This poses a significant challenge to the application of bacteriophages, as it is necessary to prevent the emergence of resistant bacteria in practical use and to find ways to overcome bacterial defense systems. Studies have found that during the interaction process, bacteriophages have also evolved anti-defense systems. Thus far, discovered phage anti-defense systems can counteract 27 out of 152 different bacterial defense families [95]. Therefore, bacteria with defense systems can be utilized to isolate phages possessing anti-defense mechanisms. Moreover, the efficiency of co-evolution can be accelerated through human intervention. Srikant et al. demonstrated that after co-culturing the T4 bacteriophage with its host bacteria for five rounds, the copy number of the *tifA* gene increased, allowing it to evade the host immune system [96].

## 6. Outlook of Phage Applications

### 6.1. Phage Cocktail

Phage cocktails are often composed of several phages with complementary host ranges, targeting a single species or multiple species. The main goal is to address the narrow host range of individual phages. When designing a phage cocktail, it is not a random combination of various phages with lytic activity. Factors such as phage stability, burst size, latent period duration, and the presence of virulence genes need to be considered. Sometimes, to avoid the emergence of resistant bacteria, phages with different infection mechanisms are chosen. These phages can target different receptors, including flagella, O antigens, capsular polysaccharides, and LPS. There have also been related studies on the application of phage cocktails in seafood. Cui et al. evaluated the preventive effect of feeding a phage mixture on ascites in turbot and found that the number of Vibrio and Edward Siella in the gut of turbot significantly decreased after feeding the phage cocktail. They demonstrated the safety of the phage cocktail through various aspects, including blood routine indicators, serum biochemical indicators, the expression of inflammatory cytokine-related genes, and changes in gut bacteria after feeding the phage cocktail [97]. The microbial communities in seafood are often relatively complex, and in most cases, the use of a single phage does not achieve good results and is not suitable for long-term use. Phage cocktails can effectively solve this problem and have great development potential.

### 6.2. Designing New Phages

A key advantage of phage therapy is the ability to isolate natural phages from environmental sources. However, the safety, therapeutic efficacy, and host specificity of natural phages present significant challenges for practical applications. These limitations can currently be addressed through genome editing to modify phage characteristics or by employing synthetic genomics to design phages tailored to specific needs. Dunne et al. re-engineered the host range of Listeria phage PSA through structure-guided design of chimeric receptor-binding proteins (RBPs). They inserted a second rbp allele, which targets different Listeria serotypes, into the genome of Listeria phage PSA, thereby expanding the host range of phage PSA [98]. With the emergence of precise gene-editing technologies and the maturation of synthetic genomics, it is anticipated that many phage genomes will be engineered effectively in the future [99].

### 6.3. Bacteriophages Encapsulation

Phage encapsulation generally involves enclosing bacteriophages within microcapsules made from natural or synthetic polymeric materials. The primary objective of this process is to preserve bacteriophage stability, enabling them to withstand adverse environmental conditions. Moreover, it enhances targeting capabilities, facilitating the controlled release of phages at specific sites or designated times [100]. Spray drying, spray freeze-drying, freeze-drying, extrusion dripping, emulsion, and polymerization techniques are currently the most commonly used methods for phage encapsulation [101]. Silva Batalha et al. employed various hydrocolloids to encapsulate the UFV-AREG1 bacteriophage using the extrusion method. Their study demonstrated an encapsulation efficiency of 99%. In simulated gastric fluid, the combination of alginate and whey protein provided effective protection for the phages, whereas in simulated intestinal fluid, around 50% of the bacteriophages were released within 10 min [102]. Choi et al. encapsulated T4 bacteriophages using a maltodextrin and trehalose blend and investigated its effect on phage viability. They found that the blend of 11% maltodextrin and 4% trehalose was the most effective, maintaining phage viability for six weeks at room temperature with a relative humidity of 22.6% [103]. The high cost of phage encapsulation remains a significant drawback. However, with the discovery of novel encapsulation materials and advancements in encapsulation techniques, production costs are anticipated to gradually decrease. Moreover, the added value of the final product may offset and compensate for the initially high costs [100].

## 7. Conclusions

The application of bacteriophages in seafood primarily resides in their capacity as biocontrol agents, efficiently regulating the proliferation of pathogenic bacteria and augmenting the safety and shelf life of seafood. The specificity and efficacy of bacteriophages render them an optimal alternative to conventional control measures, diminishing the reliance on antibiotics and alleviating the emergence of antibiotic resistance. Moreover, the diversity and adaptability of bacteriophages establish a robust foundation for their extensive application within the seafood industry. Researchers are investigating the integration of bacteriophages with other biotechnologies, including probiotics and biofilm technology, to bolster disease resistance and mitigate bacterial infections. Nevertheless, the development of bacteriophages encounters challenges, including the assurance of their stability and effectiveness in complex marine environments. Research teams are actively addressing these challenges, including the optimization of phage selection and storage methodologies to enhance their resilience. In the future, the tailored development of bacteriophages, in conjunction with advanced genetic engineering and synthetic biology, is anticipated to yield enhanced efficiency and specificity, thereby presenting new developmental opportunities within the seafood industry.

## Figures and Tables

**Table 1:** Application of bacteriophages in microbial control.

Target Organism	Phages	Seafood	Temp	Results and Observation	Reference
*Vibrio parahaemolyticus*	BP14	Shrimp	25 °C	A bacteriophage strain BP14, suitable for use in aquaculture, was isolated.	[35]
	VPT02	Brine shrimp, RTE raw fish flesh slices	5 °C or 25 °C	The survival rate of brine shrimp increased from 16.7% to 46.7%, and the number of *V. parahaemolyticus* in RTE raw fish flesh slices was reduced by up to 3.9 log.	[36]
	PVP1 and PVP2	Sea cucumbers	18–20 °C	Phage feeding treatment increased the survival of sea cucumbers infected with *V. parahaemolyticus* VP-ABTNL, and there is no effect on the normal growth of sea cucumbers.	[37]
	CAU_VPP01	Squid and mackerel	4 °C	At MOI 10, phage CAU_VPP01 effectively reduced biofilm proliferation and biomass volume, thickness, and roughness at 4 °C.	[38]
	VPG01	Paralichthys olivaceus	25 °C	When a cutting board and a seafood item were treated with VPG01, the pathogen load was significantly decreased.	[9]
*Vibrio vulnificus*	VVP001	Abalone	4 °C	VVP001 steadily inhibited *V. vulnificus* MO6-24/O up to 8 h.	[39]
*Vibrio harveyi*	VB_VhaP_Vh-5 and VB_VhaP_Vh-8	turbot Scophthalmus maximus	18 °C	Phage feeding could improve the survival rate of turbot infected by *V. harveyi* VH5 and had no effect on the normal growth of turbot.	[40]
*Listeria monocytogenes*	vB-LmoM-SH3-3	Raw salmon	4 °C	The application of phage SH3-3 in ready-to-eat salmon may reduce *Listeria* counts by 4.54 log within 72 h.	[41]
	Listex™ P100	Rakfisk	7 °C or 8 °C	An average of 0.9 log reduction was observed throughout the fermentation period.	[42]
*Salmonella*	SLMP1	Raw Salmon Fillets and Scallop	4 °C, 15 °C or 25 °C	The *Salmonella* counts of both inoculum levels on samples could be reduced below the detection limit or maintained at a low level by phage SLMP1 during storage at 4 °C.	[43]
	phSE-2 and phSE-5	Cockle	16 °C	The application of single phage suspensions of phSE-2 and phSE-5 and phage cocktail phSE-2/phSE-5 can be successfully employed to inactivate *Salmonella* spp. in cockles during depuration.	[44]

**Table 2:** Limitations of phage applications and possible solutions.

Problem	Limitations	Solutions	Reference
Public acceptance	The use of phages is still affected by ignorance, fear, and doubts.	To improve the dissemination of knowledge about phages.	[70]
Stability	Most of the phages tend to lose their stability at cold storage.	Phage can be encapsulated using natural materials to maintain phage stability.	[71]
Safety	Phages can carry and transmit drug resistance genes.	Ensure that a virulent phage is used before application.	[72,73]
Resistance	Bacteria may rapidly develop resistance (even when in cocktails).	Continuous isolation and purification of novel, safe, and highly virulent phages.	[74,75,76]
Efficacy	Need of high multiplicity of infection (MOI) due to lower burst size of the phage.	Phage can be coupled with other bactericidal technologies.	[77,78,79]

## Data Availability

The original contributions presented in this study are included in the article; further inquiries can be directed at the corresponding author.
